# Sex differences in cardiovascular risk management for people with diabetes in primary care: a cross-sectional study

**DOI:** 10.3399/bjgpopen19X101645

**Published:** 2019-05-29

**Authors:** Marit de Jong, Rimke C Vos, Rianneke de Ritter, Carla J van der Kallen, Simone J Sep, Mark Woodward, Coen DA Stehouwer, Michiel L Bots, Sanne AE Peters

**Affiliations:** 1 PhD Student, Julius Center for Health Sciences and Primary Care, University Medical Center Utrecht, Utrecht University, Utrecht, The Netherlands; 2 Assistant Professor, Department of Public Health and Primary Care / LUMC-Campus, Leiden University Medical Center, The Hague, The Netherlands; 3 Assistant Professor, Julius Center for Health Sciences and Primary Care, University Medical Center Utrecht, Utrecht University, Utrecht, The Netherlands; 4 PhD student, Department of Internal Medicine and Cardiovascular Research Institute Maastricht, Maastricht University Medical Center, Maastricht, The Netherlands; 5 PhD student, CARIM School for Cardiovascular Diseases, Maastricht University, Maastricht, The Netherlands; 6 Assistant Professor, Department of Internal Medicine and Cardiovascular Research Institute Maastricht, Maastricht University Medical Center, Maastricht, The Netherlands; 7 Assistant Professor, CARIM School for Cardiovascular Diseases, Maastricht University, Maastricht, The Netherlands; 8 Senior Researcher, Department of Internal Medicine and Cardiovascular Research Institute Maastricht, Maastricht University Medical Center, Maastricht, The Netherlands; 9 Senior Researcher, CARIM School for Cardiovascular Diseases, Maastricht University, Maastricht, The Netherlands; 10 Professor, The George Institute for Global Health, University of Oxford, Oxford, UK; 11 Professor, The George Institute for Global Health, University of New South Wales, Sydney, Australia; 12 Professor, Department of Epidemiology, Johns Hopkins University, Baltimore, USA; 13 Professor, Department of Internal Medicine and Cardiovascular Research Institute Maastricht, Maastricht University Medical Center, Maastricht, The Netherlands; 14 Professor, CARIM School for Cardiovascular Diseases, Maastricht University, Maastricht, The Netherlands; 15 Professor, Julius Center for Health Sciences and Primary Care, University Medical Center Utrecht, Utrecht University, Utrecht, The Netherlands; 16 Associate Professor, Julius Center for Health Sciences and Primary Care, University Medical Center Utrecht, Utrecht University, Utrecht, The Netherlands; 17 Associate Professor, The George Institute for Global Health, University of Oxford, Oxford, UK

**Keywords:** primary health care, sex characteristics, diabetes mellitus, cardiovascular risk management, general practice

## Abstract

**Background:**

Diabetes is a stronger risk factor for cardiovascular complications in women than men.

**Aim:**

To evaluate whether there are sex differences in cardiovascular risk management in patients with diabetes in primary care.

**Design & setting:**

A cross-sectional study was undertaken using data from 12 512 individuals with diabetes within the Dutch Julius General Practitioners Network (JGPN) from 2013.

**Method:**

Linear and Poisson regression analyses were used to assess sex differences in risk factor levels, assessment, treatment, and control.

**Results:**

No sex differences were found in HbA1c levels and control, while small differences were found for cardiovascular risk management. Blood pressure levels were higher (mean difference [MD] 1.09 mmHg; 95% confidence intervals [CI] = 0.41 to 1.77), while cholesterol levels (MD -0.38 mmol/l; 95% CI = -0.42 to -0.34) and body mass index ([BMI] MD -1.79 kg/m^2^; 95% CI = -2.03 to 1.56) were lower in men than women. Risk factor assessment was similar between sexes, apart from high-density lipoprotein cholesterol (HDL-c), which was more commonly assessed in women (risk ratio [RR] 1.16; 95% CI = 1.13 to 1.20). Among those with a treatment indication for prevention, women with cardiovascular disease (CVD) were less likely to receive lipid-lowering drugs (RR 0.84; 95% CI = 0.76 to 0.93) than men, while women without CVD were more likely to receive lipid-lowering drugs (RR 1.16; 95% CI = 1.04 to 1.2). Among those treated, women were more likely to achieve systolic blood pressure (SBP) control (RR 1.06; 95% CI = 1.02 to 1.10) and less likely to achieve low-density lipoprotein cholesterol (LDL-c) control (RR 0.88; 95% CI = 0.85 to 0.91) than men.

**Conclusion:**

In this Dutch primary care setting, sex differences in risk factor assessment and treatment of people with diabetes were small. However, women with diabetes were less likely to achieve control for LDL-c and more likely to achieve blood pressure control than men with diabetes.

## How this fits in

Women with diabetes bear a greater excess risk for the development of major cardiovascular complications than men. So far, no clear explanation for this greater excess risk in women is known, although sex differences in cardiovascular risk management may be involved. Therefore, the present study assessed differences in cardiovascular risk management between men and women with diabetes. Although sex differences in cardiovascular risk management were found to be limited, healthcare providers should be aware of the sex differences between men and women with diabetes, and cardiovascular risk management can be improved on multiple aspects for both sexes.

## Introduction

Diabetes mellitus is one of the most prevalent chronic disorders globally, with an estimated prevalence of 425 million affected individuals in 2017.^1^ Individuals with diabetes are 2–3 times more likely to develop CVD compared with individuals without diabetes. Large-scale meta-analyses have demonstrated that the excess risk of major cardiovascular complications associated with diabetes is substantially greater in women than men.^2,3^ So far, no clear explanation for the greater excess risk of major cardiovascular complications in women has been identified, although sex differences in cardiovascular risk management may be involved.^4^ Guideline-recommended management for the prevention and delay of cardiovascular complications in individuals with diabetes focuses on optimising lifestyle factors, including smoking behaviour, physical activity, diet, and weight control, and adequate management of blood pressure, cholesterol, and glucose levels.^5,6^

Previous studies have reported mixed findings regarding the presence, magnitude, and direction of sex differences in cardiovascular risk management for people with diabetes.^7–12^ For example, the National Diabetes Audit in the UK demonstrated that women were less likely than men to receive annual tests for cardiovascular risk factors and to achieve treatment targets.^7^ In contrast, a large cross-sectional study among 18 000 men and women with diabetes in the US showed that women with diabetes were more likely than men to receive annual dilated eye exams and blood pressure control tests, and to visit a doctor than men with diabetes. Moreover, while the magnitude of the sex difference in the complications of diabetes varies by age, it remains unknown whether any difference in cardiovascular risk management is age-specific.^13^

Therefore, this study evaluated the presence of sex differences in cardiovascular risk management for individuals with diabetes across different age groups in a large Dutch population attending primary care.

## Method

Routinely collected data from the JGPN from 2013 were used. The JGPN is a large, ongoing, dynamic cohort of primary care patients that anonymously extracts routine healthcare data from electronic primary care records at one of the included general practices in Utrecht (the Netherlands) and its vicinity, as detailed elsewhere.^14^ All individuals in care at one of the JGPN practices are included in the JGPN cohort. Adult individuals were included in this study if they were previously diagnosed with diabetes mellitus (International Classification of Primary Care [ICPC] T90 or K99_06), and had been registered at the primary care practice for ≥12 months in 2013 (further information available from the author on request).

### Data extraction

Data on cardiovascular risk factors, blood tests, physical measurements, history of cardiovascular events, and drug prescriptions were extracted from the medical records. The last available measurement in 2013 of the following cardiovascular risk factors of importance in diabetes care were included: HbA1c, SBP, diastolic blood pressure (DBP), total cholesterol (TC), LDL-c, HDL-c, and BMI. Medical history of CVD was determined according to the ICPC-1 (further information available from the author on request).^15^ Data on drug prescriptions for glucose-lowering drugs, lipid-lowering drugs, and antihypertensive drugs were coded using the anatomical therapeutic chemical classification (ATC) system (further information available from the author on request). ^16^

### Outcomes of interest

Four aspects of cardiovascular risk management were assessed by sex. First, it needed to be determined whether or not an assessment had been performed for each of the cardiovascular risk factors. Second, the difference between the sexes for the last measured value of cardiovascular risk factors in 2013 was assessed. Third, among those with a treatment indication, as detailed below, for lowering HbA1c, SBP, or LDL-c, the proportion of individuals that received pharmacological treatment was assessed. Fourth, among those receiving pharmacological treatment, the proportion of individuals that attained adequate levels according to Dutch guidelines was examined. These guidelines say that individuals at 10-year risk of CVD of >20% and with inadequate levels of SBP (>140 mmol/l) or LDL-c (>2.5 mmHg) are eligible for antihypertensive or lipid-lowering drugs, and individuals with HbA1c off target (53 mmol/mol [>7.0%]) are eligible to receive glucose-lowering drugs.^5,6^ CVD risk was assessed using the Systematic Coronary Risk Evaluation.^6^ Since all individuals in this study were previously diagnosed with diabetes, 15 years were added to their original age, as recommended by the Dutch cardiovascular risk management classification tool.^6^ Additionally, all individuals with a known history of CVD were classified as high risk (>20%).

### Statistical analysis

Sex-specific baseline characteristics are presented as *n* and percentages for categorical variables, and as means with standard deviations (SD) for continuous variables, overall and stratified by age group. Age groups were categorised as 18–39 years, 40–49 years, 50–59 years, 60–69 years, 70–79 years, 80–99 years, and also as <60 years and ≥60 years. Poisson regression analyses with robust standard errors were used to estimate women-to-men RRs and 95% CIs for analyses on sex associated with assessment, treatment, and control of cardiovascular risk factors. Linear regression analyses were used to calculate women-to-men MDs and 95% CIs for cardiovascular risk factor levels. Complete-case analyses were used throughout, and adjusted for age in the overall analyses, but not in the analyses by age group. In secondary analyses, women-to-men MDs and 95% CIs in cardiovascular risk factor levels were adjusted for drug prescriptions. Additionally, analyses were stratified according to previous history of CVD. An interaction term was added to the models for sex with age (as a continuous variable) and for sex with known history of CVD, to assess whether the effect of sex on the outcomes of interest varied with age and known history of CVD. All analyses were performed using SPSS Statistics (version 21).

## Results

For this study, routine care data were used from all 193 643 registered individuals aged ≥20 years and <100 years in care in 2013 at one of the 53 JGPN general practices. The 2013 JGPN database included 12 512 individuals (50% women) with diabetes, with a mean age of 64 years. Of those, 31% of men and 27% of women had a known history of CVD. Women were slightly older, less likely to smoke, and more likely to have a higher BMI than men (Table 1). Further information is available from the author on request.

**Table 1.** Baseline characteristics

	Men (*n* = 6276)	Women (*n* = 6236)
**Age, mean (SD)**	63.1 (12.9)	65.1 (14.1)
**Smoking status, *n* (%)**		
Current	1168 (22.3)	815 (15.4)
Never	1583 (30.2)	3069 (57.9)
Former	2489 (47.5)	1418 (26.7)
**10-year cardiovascular disease risk, *n* (%)**		
Low (<10%)	146 (3.5%)	364 (8.1%)
Intermediate (10%–20%)	173 (4.1%)	563 (12.6%)
High (>20%)	3888 (92.4%)	3548 (79.3%)
**Known history of cardiovascular disease, *n* (%)**	1968 (31.4)	1670 (26.8)
**Known history of hypertension, *n* (%)**	3625 (57.8)	3891 (62.4)
**Measurement**		
HbA1c mmol/mol, mean (SD)	54.6 (12.8)	54.0 (11.6)
HbA1c %, mean (SD)	7.2 (3.3)	7.1 (3.2)
Systolic blood pressure, mmHg, mean (SD)	137.9 (17.2)	137.5 (18.0)
Diastolic blood pressure, mmHg, mean (SD)	79.3 (10.1)	78.5 (10.3)
Total cholesterol, mmol/l, mean (SD)	4.4 (1.0)	4.7 (1.0)
LDL-c, mmol/l, mean (SD)	2.4 (0.8)	2.6 (0.9)
HDL-c, mmol/l, mean (SD)	1.2 (0.3)	1.4 (0.3)
Body mass index, kg/m^2^, mean (SD)	29.1 (4.6)	30.7 (6.0)
Glucose-lowering drugs, *n* (%)	4475 (71.3)	4209 (67.5)
Lipid-lowering drugs, *n* (%)	3966 (63.2)	3592 (57.6)
Antihypertensive drugs, *n* (%)	4139 (65.9)	4245 (68.1)

Due to missing data, not all variables included add up to *n* = 6276 for men and *n* = 6236 for women. HDL-c = high-density liproprotein cholesterol. LDL-c = low-density liproprotein cholesterol. SD = standard deviation.

### Assessment of cardiovascular risk factors

**Figure 1.**
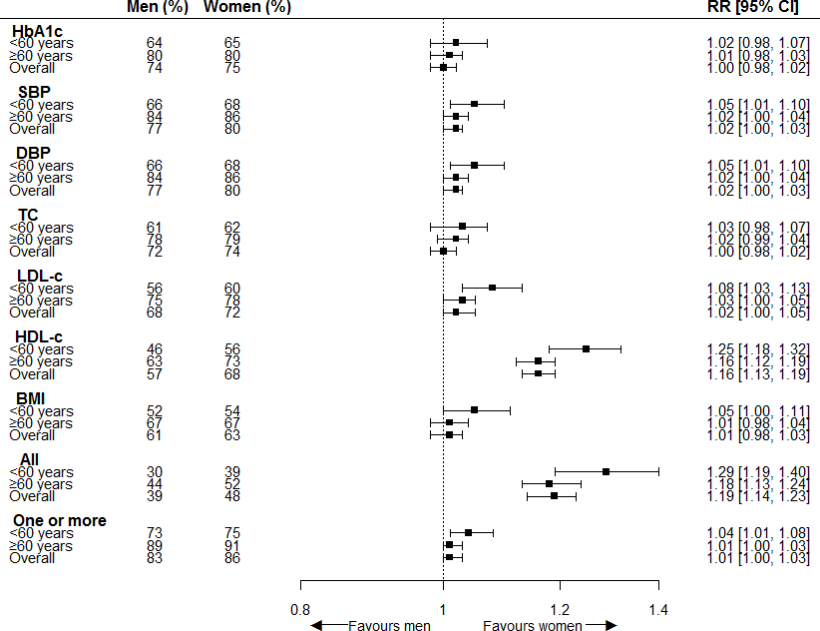
Women-to-men risk ratios for assessment of cardiovascular risk factors. The analyses are adjusted for age. Men = reference category. BMI = body mass index. CI = confidence intervals. DBP = diastolic blood pressure. HDL-c = high-density liproprotein cholesterol. LDL-c = low-density liproprotein cholesterol. RR = risk ratio. SBP = systolic blood pressure. TC = total cholesterol.

Assessment of all cardiovascular risk factors was performed in 43% of the included individuals, while assessment of the three main risk factors – HbA1c, SBP, and LDL-c combined – was performed in 63% of the included individuals. Moreover, 84% received testing for at least one cardiovascular risk factor. Blood pressure was most often assessed (79%), followed by HbA1c (75%), TC (73%), LDL-c (70%), HDL-c (62%), and BMI (43%). Testing of all cardiovascular risk factors was more likely to have been performed in women than men; the age-adjusted RR was 1.19 (95% CI = 1.13 to 1.25). HDL-c alone was more commonly assessed in women than men (RR 1.16; 95% CI = 1.13 to 1.20). Assessment of SBP, DBP, and LDL-c separately, and of one or more cardiovascular risk factors combined, was slightly greater in women than men with age-adjusted RRs of 1.02 (95% CI = 1.00 to 1.03), 1.02 (95% CI = 1.00 to 1.03), 1.02 (95% CI = 1.00 to 1.03), and 1.01 (95% CI = 1.00 to 1.05), respectively. No differences were found for assessment of HbA1c (RR 1.00; 95% CI = 0.98 to 1.02), TC (RR 1.00; 95% CI = 0.98 to 1.02), BMI (RR 1.01; 95% CI = 0.98 to 1.03), or HbA1c, SBP, and LDL-c combined (RR 1.02; 95% CI = 0.99 to 1.05), as shown in Figure 1. Although differences between age groups were small, the interaction term for sex with age as a continuous variable showed significant differences for assessment of SBP (*P* = 0.02); DBP (*P* = 0.01); LDL-c (*P* = 0.01); HDL-c (*P*<0.01); BMI (*P*<0.01); testing of all cardiovascular risk factors (*P*<0.01); assessment of HbA1c, SBP, and LDL-c combined (*P* = 0.01); and assessment of one or more cardiovascular risk factors (*P* = 0.02). No significant results were found for the interaction term of sex with CVD status. (Further information on women-to-men RRs for assessment of cardiovascular risk factors stratified for age and, separately, stratified for history of CVD is available from the author on request).

### Cardiovascular risk factor levels

**Figure 2.**
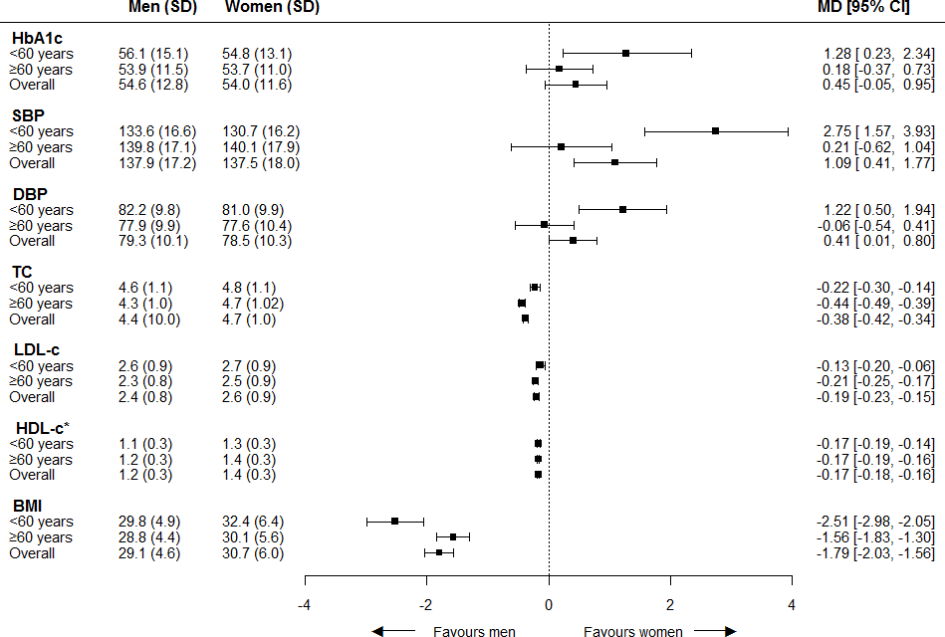
Women-to-men differences in cardiovascular risk factor levels. The analyses are adjusted for age. Women = reference category. BMI = body mass index. CI = confidence intervals. DBP = diastolic blood pressure. HDL-c = high-density liproprotein cholesterol. LDL-c = low-density liproprotein cholesterol. MD = mean difference. SBP = systolic blood pressure. SD = standard deviation. TC = total cholesterol. ^*^Increased HDL-c is in favor of women. Mean (SD) for men and women separately not adjusted for age.

Individuals included in this study had a mean HbA1c of 54 mmol/mol (7.1%), SBP of 138 mmHg, DBP of 79 mmHg, TC of 4.5 mmol/l, LDL-c of 2.5 mmol/l, HDL-c of 1.3 mmol/l, and a BMI of 30 kg/m^2^. Age-adjusted analyses showed that blood pressure was higher in men than women by 1.09 mmHg (MD; 95% CI = 0.41 to 1.77) for SBP and 0.41 mmHg (MD; 95% CI = 0.01 to 0.80) for DBP. In contrast, TC, LDL-c, HDL-c, and BMI were lower in men than women; MDs were -0.38 mmol/l (95% CI = -0.42 to -0.34) for TC, -0.19 mmol/l (95% CI = -0.23 to -0.15) for LDL-c, -0.17 mmol/l (95% CI = -0.18 to -0.16) for HDL-c, and -1.79 kg/m^2^ (95% CI = -2.03 to -1.56) for BMI. No differences were seen for HbA1c (MD 0.45 mmol/mol; 95% CI = -0.05 to 0.95), as shown in Figure 2. The results were similar after adjustment for drug prescriptions. The interaction term for sex with age as a continuous variable showed significant differences for HbA1c (*P* = 0.01), SBP (*P*<0.01), DBP (*P*<0.01), TC (*P*<0.01), LDL-c (*P* = 0.04), and BMI (*P*<0.01), showing that the effect of sex on last measured cardiovascular risk factors changes with age; although, actual differences were small. The interaction term for sex with known history of CVD showed a significant difference for DBP (*P* = 0.03), showing that the effect of sex on last measured DBP differed for individuals with CVD (MD -0.35; 95% CI = -1.09 to 0.40) and without CVD (MD 0.84; 95% CI = -0.38 to 1.31). Further information on women-to-men mean differences in cardiovascular risk factor levels stratified for age and, separately, stratified for known history of CVD is available from the author on request.

### Treatment

**Figure 3.**
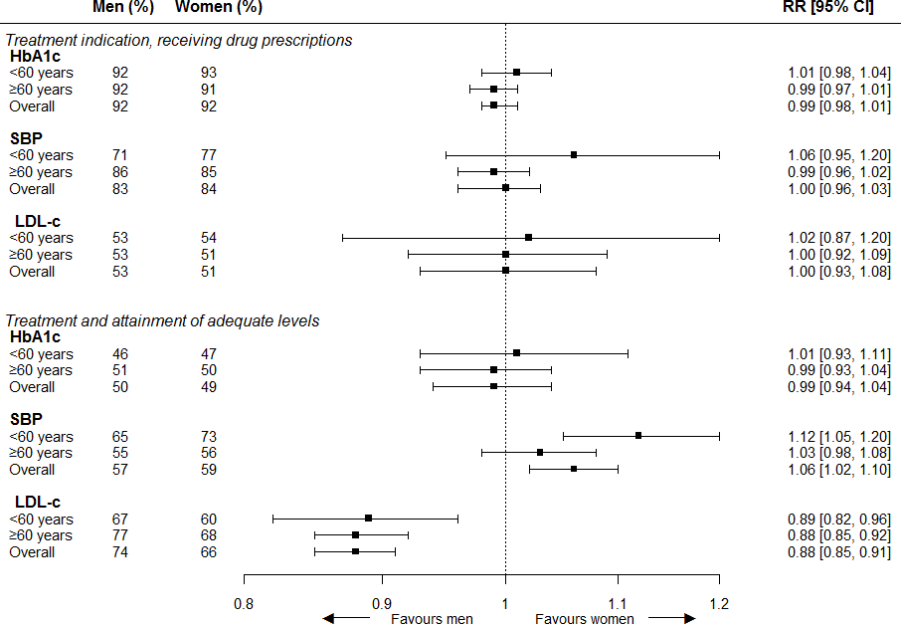
Women-to-men risk ratios for treatment and control of cardiovascular risk factors. The analyses are adjusted for age. Men = reference category. CI = confidence intervals. LDL-c = low density lipoprotein cholesterol. RR = risk ratio. SBP = systolic blood pressure.

Among those with a treatment indication for receiving drugs, 92% received glucose-lowering drugs when indicated, 84% received antihypertensive drugs when indicated, and 52% received lipid-lowering drugs when indicated. No sex differences were found for receiving glucose-lowering drugs with age-adjusted RR of 0.99 (95% CI = 0.98 to 1.01), for antihypertensive drugs (RR 1.00; 95% CI = 0.96 to 1.03), and for lipid-lowering drugs (RR 1.00; 95% CI = 0.93 to 1.08), as shown in Figure 3. The interaction term for sex with age as a continuous variable showed significant difference for receiving blood-pressure lowering drugs when indicated (*P*<0.01). Sex differences in LDL-c treatment were revealed after stratification for known CVD history; women without a known history of CVD were more likely to receive lipid-lowering drugs than men (RR 1.16; 95% CI = 1.04 to 1.29), whereas women with a known history of CVD were less likely to receive lipid-lowering drugs than men (RR 0.84; 95% CI = 0.76 to 0.93*; P* for interaction <0.01). Further information on women-to-men RRs for treatment of cardiovascular risk factors stratified for age and, separately, stratified for known history of CVD is available from the author on request.

### Risk factor control

Among those receiving glucose-lowering drugs, 49% were on target (≤7%/53 mmol/mol). For those receiving antihypertensive drugs or lipid-lowering drugs, 58% and 70% were on target for SBP (≤140 mmHg) and LDL-c (≤2.5 mmol/l), respectively. Among those treated, women were more likely than men to be on target for SBP (RR 1.06; 95% CI = 1.02 to 1.10) and less likely to be on target for LDL-c (RR 0.88; 0.85 to 0.91). No sex differences were found for control of HbA1c (RR 0.99; 95% CI = 0.94 to 1.04), as shown in Figure 3. Similar results were found after stratification for known CVD history. The interaction term for sex with age as a continuous variable was significant for being on target for SBP while receiving antihypertensive drugs (*P* = 0.02) and no significant interaction term was found for history of CVD. Further information on women-to-men RRs for control of cardiovascular risk factors stratified for age and, separately, stratified for known history of CVD is available from the author on request.

## Discussion

### Summary

In this study, the presence of sex differences in cardiovascular risk management in a Dutch population of individuals with diabetes mellitus in routine primary care was assessed. It was found that only 43% of the included individuals received assessment of all cardiovascular risk factors, while 63% of the included individuals received assessment of the main cardiovascular risk factors — HbA1c, SBP, and LDL-c combined — and 83% received testing for one or more cardiovascular risk factors. Among those with a treatment indication for lowering HbA1c, 92% received glucose-lowering drugs, while only 84% and 52% of those with a treatment indication for lowering SBP or LDL-c received prescriptions for antihypertensive drugs or lipid-lowering drugs, respectively. Furthermore, among those receiving glucose-lowering drugs, antihypertensive drugs, or lipid-lowering drugs, only 49%, 58%, and 70% were on target for HbA1c, SBP, and LDL-c respectively. Sex differences in risk factor assessment and treatment were generally small and an interaction term for sex with age as a continuous variable was found to be significant for several of the analyses, although actual differences were small. Blood pressure levels were lower and cholesterol levels were higher in women than men. Among those treated, women were less likely than men to achieve adequate control for LDL-c, but more likely to achieve blood pressure control.

### Strengths and limitations

Using routinely collected data from 53 primary care practices in the Netherlands, this study provides a representative evaluation of sex differences in cardiovascular risk management among Dutch individuals with diabetes attending primary care. A limitation of using routinely collected data is that the completeness of data depends on recording practices of GPs. For example, recording of smoking status in primary care data was incomplete. While it may be that some aspects of cardiovascular risk management were performed but not recorded, there is no reason to assume that underreporting of delivered care differs between women and men. Also, diabetes is a rapidly changing field and management guidelines may have changed after the data were collected. Nevertheless, more recent guidelines have not implemented sex-specific approaches for the management, treatment, and control of diabetes. Therefore, it is anticipated that the sex differences found in this study are still valid. Moreover, no information on health care provided by healthcare professionals other than GPs was available. Hence, it may be that healthcare professionals other than the GP had conducted cardiovascular risk assessment or had prescribed drug therapy; for example, roughly 20% of individuals with diabetes in the Netherlands are referred to a specialist for specialised care. For the analyses, the authors were unable to assess whether there were meaningful differences between men and women in care provided by other healthcare professionals. For the analyses on treatment indication and drugs prescription history, it was decided to only include individuals with a treatment indication based on CVD risk score and last measured levels of SBP or LDL-c for antihypertensive drugs and lipid-lowering drugs, and last measured HbA1c levels for glucose-lowering drugs. Consequently, individuals who were on target for last measured HbA1c, SBP, or LDL-c while receiving glucose-lowering drugs, antihypertensive drugs, or lipid-lowering drugs were not included. Moreover, individuals that received glucose-lowering drugs, antihypertensive drugs, or lipid-lowering drugs, but with missing data on either CVD risk score for the analyses on SBP or LDL-c or missing data on last measured levels of HbA1c, SBP, or LDL were not included, which must be taken into account when interpreting the results.

### Comparison with existing literature

Previous studies on differences in cardiovascular risk management between men and women with diabetes have reported mixed findings.^7–12^ A study in the US including 18 000 individuals showed that women had higher odds of receiving dilated eye exams (odds ratio [OR] 1.14; 95% CI = 1.04 to 1.24), blood pressure control (OR 1.44; 95% CI = 1.13 to 1.84), and of visiting a doctor (OR 1.39; 95% CI = 1.22 to 1.58) than men, while no differences were found for testing HbA1c (OR 1.01; 95% CI = 0.89 to 1.14) and feet checked in a given year (OR 0.91; 95% CI = 0.83 to 1.00). ^8^. In contrast, a population-based study in Spain among 290 000 individuals showed that women had worse overall control of cardiovascular risk factors than men.^10^ These differences, stratified for history of CVD, were mainly evident for BMI with adjusted ORs of 0.50 (95% CI = 0.48 to 0.52) and 0.53 (95% CI = 0.52 to 0.54), and for LDL-c with ORs of 0.67 (95% CI = 0.64 to 0.70) and 0.74 (95% CI = 0.72 to 0.76), while differences in blood pressure were less evident with ORs of 0.88 (95% CI = 0.84 to 0.92) and 1.08 (95% CI = 1.06 to 1.13) for women compared with men with and without CVD respectively. In contrast, women were more likely than men to be non-smokers with adjusted ORs of 4.20 (95% CI = 3.86 to 4.58) and 4.01 (95% CI = 3.39 to 4.13) with and without CVD respectively, and no differences were found for HbA1c control.^10^ A population-based study from Italy, including 415 000 individuals, showed that women were more likely to be off target for HbA1c (OR 1.14; 95% CI = 1.10 to 1.17) in spite of insulin treatment, LDL-c (OR 1.42; 95% CI = 1.38 to 1.46) in spite of receiving lipid-lowering drugs, and BMI ≥30 kg/m^2^ (OR 1.50; 95% CI = 1.50 to 1.54), while no differences were found for blood pressure while receiving antihypertensive drugs (95% CI = 1.02; 1.00 to 1.04).^11^

The present study demonstrates that sex differences in cardiovascular risk management among patients with diabetes are relatively small in the Netherlands. Control of blood pressure, one of the biggest risk factors for CVD, was even more favourable among women than men, suggesting that differences in cardiovascular risk management alone may not fully account for the higher relative risks previously found in women compared with men.^2,3^ Other factors, such as biological differences between men and women and differences in treatment adherence, may therefore play a key role in explaining the sex differences in the cardiovascular complications conferred by diabetes. For example, it has been suggested that the metabolic state and cardiovascular risk profile of women needs to deteriorate further than in men before the transition to overt diabetes occurs, especially with regard to adiposity.^17^ A large population-wide study among 95 000 individuals in Scotland showed that women had on average a 2-point higher BMI than men at diagnosis of diabetes.^18^ Fat distribution differs by sex, with greater subcutaneous fat storage in women, on average, and greater visceral and ectopic fat storage in men. Since visceral and ectopic fat are associated with insulin resistance and development of diabetes, it has been hypothesised that men develop diabetes at lower BMI than women because women can store more fat subcutaneously before transition to visceral and ectopic tissues.^17,18^ Moreover, compared with men, women may be exposed to adverse cardiovascular risk factors for a longer period before they eventually are diagnosed with overt diabetes and receive adequate treatment.^18^ In line with this hypothesis, an Australian review and meta-analyses on the duration of pre-diabetes showed that the duration of pre-diabetes was 10.3 years in women, compared with 8.5 years in men.^19^

Inadequate adherence to cardiovascular drugs often leads to suboptimal cardiovascular risk factor control and has been associated with adverse cardiovascular outcomes.^20–22^ Several studies in the general population have suggested that adherence to statins, antihypertensive drugs, and insulin is worse in women than men.^17,18,23,24^ Nevertheless, it is unknown to what extent sex differences in drug adherence among individuals with diabetes exist and to what extent such differences, if present, may explain the greater excess cardiovascular risk in women with diabetes compared with men. Future research should, therefore, evaluate whether sex differences in medication adherence among patients with diabetes are present. Such studies should also consider possible sex differences in drug type and dosage, especially since it was observed that, given treatment, control of LDL-c was worse among women than men, but control of SBP was better among women than men.

### Implications for practice

In conclusion, the implementation of cardiovascular risk management can be improved on multiple aspects for both sexes, including assessment of cardiovascular risk factors. No sex differences in Hba1c levels and control were found, and sex differences in cardiovascular risk factor assessment and treatment were small in this population of patients with diabetes attending primary care. Nevertheless, women with diabetes were less likely to achieve control for LDL-c and more likely to achieve blood pressure control than men with diabetes. Moreover, weight loss strategies will be required to reduce the high BMI scores, of 29.1 kg/m^2^ in men and 30.7 kg/m^2^ in women, in both sexes.

This study mainly focused on screening and control of HbA1c, blood pressure, lipid spectrum, and BMI. However, while not assessed here, adequate diabetes management goes beyond the management of HbA1c alone and also involves the assessment of renal function (serum creatinine and urine albumin/creatinine ratio), smoking status, foot surveillance, and retinal screening.^25^

Further information is available on request from the author for details on the ICPC-1 codes, and for more information on the ATC codes included for each drug category.
